# Challenges and Delay in Treatment With Clozapine Due to Thrombocytopenia: A Case Study

**DOI:** 10.1192/bjo.2024.691

**Published:** 2024-08-01

**Authors:** Suresh Thapaliya, Glenda Boutell, Mudasir Firdosi

**Affiliations:** Kent and Medway NHS and Social Care Partnership Trust, Kent, United Kingdom

## Abstract

**Aims:**

Clozapine is the most effective antipsychotic medication for patients with treatment-resistant psychotic disorders. Its discontinuation can precipitate relapse that can be often challenging to treat.

**Methods:**

This is a case study of a female patient in her early 40s who is known to the mental health services with a diagnosis of schizoaffective disorder. She was admitted to acute psychiatric inpatient unit due to relapse characterised by psychotic, catatonic features and poor physical health condition due to refusal to eat and drink. She was stable on clozapine for more than a decade and had become unwell after discontinuation of clozapine in the community due to platelet count below 50 × 109/L *with normal other parameters.* Low platelet count was detected during routine monthly blood monitoring after a few years of commencing clozapine.

Whilst an inpatient, there were several trials of re-titration of clozapine which had to be withheld because of ambiguity regarding the cause of persistent thrombocytopenia. Other treatment options including alternative antipsychotics and 12 sessions of ECT were tried without any success. Haematologist opinion was sought at early stage of admission and blood investigations were done but there was delay in bone marrow biopsy due to practical issues.

The treating team re-commenced oral clozapine to which she remained initially non-compliant due to catatonic features. With advice from the specialist psychosis services a few doses of intramuscular clozapine was used to facilitate re-titration. Following regular compliance and optimisation of oral clozapine, there was significant remission of clinical symptoms, with patient returning to her baseline mental state and functioning. During the period of admission, platelet counts were closely monitored which kept fluctuating reaching sometimes below 30 × 109/L without any clear association with clozapine dose. No bleeding symptoms or signs were ever reported.

**Results:**

Clozapine is a medication with haematological side effects; however, low platelet count is very rare. This patient ultimately underwent bone marrow biopsy which established Immune thrombocytopenia. She was discharged to the community with a plan of continuing clozapine, close monitoring of blood count and regular follow-up with haematology services for further clinical management.

**Conclusion:**

Careful clinical evaluation and timely investigation is important to establish the cause for side effects before associating it with clozapine and discontinuing the treatment. This helps in ensuring continuity of clozapine in patients who clearly benefit from long-term use of clozapine.

